# Prevalence estimation of *Tropheryma whipplei* in duodenal biopsy tissues of Koreans

**DOI:** 10.1186/s12941-023-00658-z

**Published:** 2024-01-03

**Authors:** Sumi Yoon, Min Eui Hong, Soon Auck Hong, Tae-Hyoung Kim, Mi-Kyung Lee

**Affiliations:** 1 Department of Laboratory Medicine, Chung-Ang University College of Medicine, Seoul, Korea; 2 Department of Pathology, Chung-Ang University College of Medicine, Seoul, Korea; 3 Department of Urology, Chung-Ang University College of Medicine, Seoul, Korea

**Keywords:** *Tropheryma whipplei*, Prevalence, Duodenal biopsy tissue, Korean, Real-time PCR, 16S–23S rRNA intergenic spacer, *hsp65*, *Dig15*

## Abstract

Whipple’s disease caused by *Tropheryma whipplei* is difficult to diagnose because of a broad spectrum of manifestations and non-specific clinical signs. In the current global era, the incidence of duodenal infection/inflammation caused by *T. whipplei* in Korea may has been underestimated. Here we estimated the prevalence of *T. whipplei* in duodenal biopsy tissues of Koreans using real-time PCRs (RT-PCRs). A total of 252 duodenal biopsy tissues were collected from Korean patients who underwent esophagogastroduodenoscopy and duodenal biopsy. DNA extracted from the duodenal biopsy tissues was analyzed using three RT-PCRs targeting *T. whipplei*-specific regions of the 16S–23S rRNA intergenic spacer, *hsp65*, and *Dig15* in parallel. In the samples positive in RT-PCRs, direct sequencing was performed for each RT-PCR target. The prevalence of *T. whipplei* was estimated based on the RT-PCR and sequencing results. Among the analyzed samples, *T. whipplei* was not detected. The prevalence of *T. whipplei* in duodenal biopsy tissues of Koreans was estimated to be less than 0.4%. This is the first study to attempt to detect *T. whipplei* in duodenal biopsy tissues of Koreans and estimate its prevalence. Our findings infer that while *T. whipplei* carriers exist in Korea, the incidence of duodenal infection/inflammation caused by *T. whipplei* is extremely rare.

## Background

Whipple’s disease (WD) is a multisystemic infectious disease caused by *Tropheryma whipplei*, which, despite being rare, can be life-threatening if untreated [1]. *T. whipplei* infections exhibit a broad spectrum of manifestations, encompassing classic WD, localized chronic infections, acute infections, and asymptomatic carriage [1, 2]. Diagnosis of *T. whipplei* infections using clinical and microbiological approaches remains difficult [1]. *T. whipplei* infections present with highly polymorphic and non-specific clinical signs, and even asymptomatic carriers exist [1]. Additionally, *T. whipplei* is a complex and challenging bacterium to cultivate in clinical laboratories, requiring a specific medium and supplements [1, 3]. For this reason, histopathology and polymerase chain reaction (PCR) are commonly employed as routine methods for diagnosing *T. whipplei* infections [1, 3]. Histologically, the presence of foamy macrophages and bacteria can be observed in infected lesions of the small intestine using periodic acid-Schiff and hematoxylin-and-eosin staining [1, 2]. PCR is considered more sensitive and specific than other methods [1]. PCR targeting a *T. whipplei*-specific gene on non-invasive samples (e.g. saliva and stools) can serve as a suitable screening method [1, 2]. Positive PCR results in saliva and stool samples can occur even in healthy carriers and do not necessarily indicate classic WD, which is a spectrum of *T. whipplei* infections [2]. Thus, a second PCR on invasive samples (e.g. duodenal biopsy tissue) and histopathology should be performed to assure the diagnosis of classic WD [1, 2].

Classic WD is rare, but its cause, *T. whipplei*, is an intestinal bacterium that is relatively commonly detected in the saliva and stools of healthy carriers [4]. The prevalence of *T. whipplei* has been reported to vary from 1.5% to as high as 48% depending on age, region, occupational exposure, and underlying disease [4–11]. In Europe, *T. whipplei* was detected at a prevalence of up to 4% in the stools of healthy carriers, but at a higher prevalence of 12–25% in the stools of sewer workers and patients with liver cirrhosis [5, 6]. In Senegal and Gabon, *T. whipplei* was detected in the stools of healthy carriers at a high prevalence of 31.2% and 19.6%, respectively [7, 8]. Only a few studies have been conducted in Korea to detect *T. whipplei* DNA in various samples such as joint fluid, saliva, gastric juice, and stool samples [3, 12–14]. In our previous study, we found the presence of *T. whipplei* in the stools of Korean patients with diarrhea [3]. To the best of our knowledge, no previous study in Korea has sought to detect *T. whipplei* in the biopsy tissue of human intestine known to be a natural niche for the bacterium [1]. WD has never been reported in Korea, which may be an underestimate of its incidence in the current global era, considering the nature of *T. whipplei* known to be transmitted from fecal to oral and from oral to oral [1, 3]. In this study, we aimed to estimate the prevalence of *T. whipplei* in duodenal biopsy tissues of Koreans using real-time PCRs (RT-PCRs) to determine whether the incidence of duodenal infection/inflammation caused by the bacterium has been underestimated in Korea.

## Methods

This retrospective study was conducted from February to May, 2023 in Chung-Ang University Medical Center (CAUMC), Seoul, Korea. The study protocol was approved by the Institutional Review Board (IRB) of CAUMC (2301-004-537). Informed consent from the study subjects was waived according to the IRB policy because the study used residual samples after the requested examination was performed. A total of 252 duodenal biopsy tissues were randomly collected among the samples obtained from Korean patients (median age: 57 years, interquartile range: 42–68 years) who underwent esophagogastroduodenoscopy (EGD) and duodenal biopsy for a routine medical check-up or an examination for gastrointestinal symptoms at CAUMC from January 2021 to May 2022. None of the patients were diagnosed with WD before, during, or after the hospital visit. The duodenal tissues were biopsied from visible lesions on EGD (Table 1). The duodenal biopsy tissues were stored as a paraffin-embedded block and were deparaffinized using xylene. DNA was extracted from the tissues using the QIAamp DNA Mini kit (Qiagen, Hilden, Germany) according to the manufacturer’s instructions and stored at −70°C until analysis. The data were analyzed anonymously.

**Table 1:** Characteristics of the study population (n = 252)

Characteristics	
Age (yrs), median (IQR)	
Total	57 (42–68)
Female	55 (30–68)
Male	58 (44–68)
Sex, n (%)	
Female	91 (36.1)
Male	161 (63.9)
Duodenal biopsy finding, n (%)	
Inflammatory condition	217 (86.1)
Chronic duodenitis/inflammation	159 (63.1)
Non-specific duodenitis/inflammation	44 (17.5)
Focal duodenitis	14 (5.5)
Non-inflammatory condition	35 (13.9)
Heterotopic gastric mucosa	14 (5.5)
Gastric metaplasia	6 (2.4)
Brunner’s gland hyperplasia	5 (2.0)
Others^*^	10 (4.0)

Abbreviations: IQR, interquartile range; n, number; yrs, years

^*^Others include hyperplastic polyp (n = 4), tubular adenoma (n = 3), fundic gland polyp (n = 1), lymphangioma (n = 1), and melanosis duodeni (n = 1).

RT-PCRs for *T. whipplei* were performed on the ABI 7500 Real-Time PCR System (Thermo Fisher Scientific, Waltham, MA, USA) referring to our previous publication [3]. All samples were analyzed using three RT-PCRs targeting *T. whipplei*-specific regions of the 16S–23S rRNA intergenic spacer (ITS), heat shock protein 65 (*hsp65*), and *Dig15* gene segments from the Wnt1-inducible signaling pathway family protein in parallel. The primers for each target were used as previously described [3]. The β-subunit of RNA polymerase (*rpoB*) was not used as a target of RT-PCR in this study because it showed the lowest performance for *T. whipplei* detection in our previous study [3]. Recombinant plasmids (pMG-Amp, Macrogen, Seoul, Korea) with each target sequence and distilled sterile water were used as positive and negative controls, respectively [3]. Interpretation of a positive RT-PCR result relied on the fulfillment of the following criteria: (1) the fluorescence emitted from the target sequence surpassed that of the background signal, and (2) the melting peak aligned with the melting temperature (*T*_m_) of each positive control, with a tolerance of ± 1°C. In parallel, RT-PCR targeting the human β-actin gene was performed to verify the quality of DNA extracted from the tissue [15, 16]. The detection of the human β-actin gene indicated successful extraction of DNA and the absence of PCR inhibitors [16].

**Table 2:** Results of RT-PCR and sequencing for *Tropheryma whipplei*

No. of sample	RT-PCR			Sequencing	
	16S–23S rRNA ITS	*hsp65*	*Dig15*	Positive	Negative
248	N	N	N	-	-
3	N	N	P	0	3
1	N	P	N	0	1

Sequencing analysis was performed for each RT-PCR target in the RT-PCR positive samples.

Abbreviations: ITS, intergenic spacer; N, negative; P, positive; RT-PCR, real-time PCR

In the samples positive for *T. whipplei* by RT-PCR, direct sequencing analysis was performed for each RT-PCR target using the ABI 3730*xl* DNA Analyzer (Thermo Fisher Scientific) to validate the presence of *T. whipplei*. The products of each RT-PCR were sequenced using the same forward primers as those employed for amplification. Sequence similarity searches were performed using the Basic Local Alignment Search Tool (BLAST) at the NCBI website (http://www.ncbi.nlm.nih.gov). The prevalence of *T. whipplei* in duodenal biopsy tissues of Koreans was estimated based on the results of RT-PCR and sequencing analysis. Statistical analysis was performed using MedCalc Statistical Software (version 20.109; MedCalc Software, Ostend, Belgium).

## Results and discussion

Among the analyzed samples, one sample showed a positive result in the RT-PCR targeting *hsp65* and three samples showed a positive result in the RT-PCR targeting *Dig15*. However, all four of these samples were confirmed to be *T. whipplei*-negative by sequencing (Table 2). By integrating the findings of RT-PCR and sequencing, we established that *T. whipplei* was not detected in any of the 252 included samples. Thus, the prevalence of *T. whipplei* in duodenal tissues of Koreans biopsied from visible lesions on EGD was estimated to be less than 0.4%. This is a lower estimate than the previously reported prevalence of *T. whipplei* in the stools of Korean patients with diarrhea (1.4%) [3]. Considering the high sensitivity and low detection limits of RT-PCRs as previously identified, our present findings are highly convincing [3].

It is possible that *T. whipplei* DNA was present in the samples at levels below the detection limit of the RT-PCRs used in this study. Alternatively, various factors may have reduced the sensitivity of RT-PCR. Since the previous and present studies differed in that the samples were stools and duodenal biopsy tissues, respectively, we used a kit appropriate for DNA extraction from each sample. Nevertheless, the use of different kits to extract DNA may influence the sensitivity of the test, especially for low bacterial biomass samples [17]. In addition, while the stools in the previous study were fresh materials, the duodenal biopsy tissues was the paraffin-embedded materials. The PCR assays on paraffin-embedded materials require optimization because the detection rates of PCR may be reduced [18, 19]. DNA extracted from *T. whipplei*-infected tissue would be appropriate as a positive control for PCR. However, this study has the limitation that recombinant plasmids were used as a positive control because it was difficult to obtain a standard strain of *T. whipplei* or *T. whipplei*-infected tissue. Instead, the quality of DNA extracted from the tissue was verified by parallel RT-PCR targeting the human β-actin gene, and the target gene was detected in all samples.

Four samples showed false-positive results by RT-PCRs. One sample with a false-positive result for *hsp65* showed mixed peaks on sequencing, for which no significant similarity was found in the BLAST search. Among the three samples with false-positive results for *Dig15*, one was confirmed as a noise signal and one as mixed peaks of *Palisada intermedia* with 86.0% identity and *Laurencia catarinensis* with 85.6% identity. The last one was confirmed as *Homo sapiens* with 97.3% identity. These sequencing results obtained from non-specific PCR products offer insights for refining primer designs to further increase the specificity of the PCR assay. In addition, the discrepancy between RT-PCR results highlights the credibility of using multiple PCR targets rather than a single target.

Our previous and present findings collectively suggest that *T. whipplei* exists in the intestine of Koreans, but may be merely a passenger. In carriers, *T. whipplei* has been detected at a high rate in stool samples, but only a relatively low rate in intestinal biopsy samples [20]. Due to differences in sample types, the prevalence in this study using duodenal biopsy tissues could be different than in our previous study using stool samples. In addition, the obtained results could be attributed to sampling bias resulting from the uneven distribution of the bacterium within the intestine [1, 3]. To address potential sampling bias, it is recommended to obtain multiple samples from different sites of the intestine [1]. The samples in this study were limited in that biopsy tissue could only be obtained from the duodenum and not from other parts of the small intestine. Although the samples were not obtained from multiple sites, they were obtained from sites that had a relatively high probability of bacterial presence, specifically from abnormal lesions that were determined to require biopsy based on endoscopic findings. Our findings indicate that *T. whipplei* is by no means a common cause of duodenal infection/inflammation in Korea. However, there is still the possibility of infection in parts of the small intestine other than the duodenum.

In this study, the majority of samples were obtained from adults. The intestinal colonization of *T. whipplei* has been shown to have a higher prevalence in children and adolescents than in adults, indicating an age-dependent presence [3, 8, 21]. It has been reported that the prevalence of antibodies against *T. whipplei* was higher in adults than in children, indicating the possibility of acquired immunity to *T. whipplei* infection [22]. Repeated reinfection and/or other host factors (e.g. immunity) appear to be required to maintain *T. whipplei* colonization in adults [21]. The host’s immune system is an important predisposing factor for *T. whipplei* infections, and the bacterial colonization alone is not sufficient to cause WD [1]. Because this study included subjects who visited the CAUMC for a routine medical check-up and was limited in obtaining information on their medical records, host factors such as underlying health conditions and drug use could not be considered. Further studies are needed to detect *T. whipplei* in a larger number of intestinal biopsy samples from Korean patients across a wider age range, taking host factors into account.

In conclusion, this is the first study to attempt to detect *T. whipplei* in duodenal biopsy tissues of Koreans and estimate its prevalence. *T. whipplei*, which had been detected at a prevalence of 1.4% in the stools of Koreans, was not detected in the duodenal biopsy tissue of the Koreans analyzed in this study. Our findings infer that while *T. whipplei* carriers exist in Korea, the incidence of duodenal infection/inflammation caused by *T. whipplei* is extremely rare, which makes it reasonable that WD has not been reported in Korea.

## Data Availability

The data used and analyzed during this study are available from the corresponding author on reasonable request.

## Abbreviations

WD: Whipple’s disease
*T. whipplei*: *Tropheryma whipplei*
PCR: polymerase chain reaction
RT-PCR: real-time PCR
CAUMC: Chung-Ang University Medical Center
IRB: Institutional Review Board
EGD: esophagogastroduodenoscopy
ITS: intergenic spacer
*hsp65*: heat shock protein 65
*rpoB*: the β-subunit of RNA polymerase
*T*_*m*_: melting temperature
